# Work-family conflict and salespeople deviant behavior: the mediating role of job stress

**DOI:** 10.1016/j.heliyon.2022.e10881

**Published:** 2022-10-07

**Authors:** Yu-Te Tu, Jovi Sulistiawan, Dian Ekowati, Hanif Rizaldy

**Affiliations:** a Department of Business Administration, Asia Management College, Asia University, Taiwan; b Department of Management, Faculty of Economics and Business, Universitas Airlangga, Indonesia

**Keywords:** Job stress, Salesperson, Work-family conflict, Workplace deviant behavior

## Abstract

The salesperson is a job that is prone to deviant behavior. Prior studies addressed that deviant behavior is a behavioral stress response. Although numerous studies have demonstrated the relationship between job stress and deviant behavior, the clarity of the job stress mechanism remains limited. Drawing from coping theory, we proposed that job stress acts as a critical mechanism in linking the association between work-family conflict and salesperson deviant behavior. The objectives of this study are threefold. First, this study investigates the relationship between work-family conflict and job stress. Second, this study examines how job stress determines the likelihood of a salesperson engaging in deviant behavior. Last, this study investigates the mediating mechanism of work-family conflict and job stress. Using an online survey of salespeople in Indonesia, we received 321 data and employed a partial least square to test our proposed hypotheses. The results of this study confirm all hypotheses. The implications for managers regarding the result of this study is encouraging managers to establish and implement family-friendly policies which can diminish the level of stress and will decrease the likelihood of salespeople engaging in deviant behavior. Our study offers a significant contribution to the body of knowledge by clarifying the mediating role of job stress.

## 1 Introduction

Employers' primary concern in any organization is employee work behavior (Sabeen and Arshad, 2019). Deviant workplace behavior is anti-normative and can have negative consequences for people, communities, and organizations (Alias et al., 2013). Deviant workplace behavior is defined as voluntary activity on the part of organizational members that undermines organizational standards and jeopardizes the organization’s and members' well-being (Darrat et al., 2016; Jelinek and Ahearne, 2006; Robinson and Bennett, 1995). The study’s initial finding is based on a Gallup survey conducted on November 4, 2017. This survey discusses the amount of honesty and ethics in specific professions, demonstrating that dishonest behavior occurs in a variety of different forms of labor. According to the poll, the salesperson is a job that is prone to deviant behavior. The study of deviant workplace behavior is critical in the field of sales because the costs associated with deviant workplace behavior are higher and have a direct effect on corporate revenues (Mount et al., 2006; Swimberghe et al., 2014). These expenditures may be more significant because the salesperson functions as a boundary spanner for the business. Unlike other occupancy, salespeople, in their capacity as boundary spanners, are not only associated with internal aspects of the company but also with external components such as clients. Forkmann et al. (2022) posited that salespeople, in their capacity as boundary spanners, contribute to strategy implementation, and the salesperson role is one of the most critical of all organizational tasks involved in strategy implementation.

According to studies on deviance, it is a behavioral response to job stress (Diefendorff and Mehta, 2007; Junaedi and Wulani, 2021; Swimberghe et al., 2014). Stress is a consequence of disagreement and has been demonstrated to be a significant antecedent to deviant behavior (Swimberghe et al., 2014). When employees are unable to advance their roles or are faced with higher workloads, they experience intense stress and look for alternative means of reestablishing balance in their life. Amongst numerous stressors, conflict at the work-family interface is a crucial one. Numerous studies have demonstrated a relationship between work-family conflict and employee attitudes and deviant behavior against the organization and coworkers (Darrat et al., 2010, 2017; Swimberghe et al., 2014). Despite existing evidence, there were no studies examining the association between work-family conflict and deviant behavior. What is absent is a conceptual framework for comprehending how work-family conflict drives deviant behavior.

According to coping theory (Lazarus and Folkman, 1984), increased levels of stress are connected with a rise in deviant behavior by salespeople in response to organization unfairness (Schwepker and Dimitriou, 2021; Swimberghe et al., 2014). Job stress can lead to self-initiated coping techniques by salespeople. Empirical investigations of employee-related job stress coping techniques shed light on the strategy implemented by employees who value their work role to minimize the difference between work and personal life. The focus of this research is to determine whether the mediating mechanism of stress caused by work-family conflict increases an individual’s proclivity for deviant behavior. This study contributes to the coping theory by applying it to the field of job stress by analyzing the mediation mechanism.

The purposes of this study are as follows. First, we examine the effect of work-family conflict on job stress. Second, we investigate how job stress determines the salesperson’s deviant behavior. Third, we conduct mediating analysis to investigate the mechanism of job stress in mediating the link between work-family conflict and all types of salesperson deviant behavior. To attain the objectives of this study, we employed partial least squares (PLS). PLS is employed to assist us in ensuring data quality by conducting validity and reliability assessments and testing our proposed hypotheses.

This study contributes to the literature on several points. First, to our best knowledge, few studies examined WFC using both directions of the conflict, work interfering with family (WIF) and family interfering with work (FIW), in the context of the salesperson. Second, we focus on the physical and psychological health of stressed salespeople, whereas prior studies only considered job stress as a stressor. Third, we employed specific deviant behavior that fit with the context of the salesperson, namely front-line deviant behavior.

The remainder of this study is as follows, the literature review and proposed hypotheses are presented in section 2. Section 3 provides the methodology utilized in this study. The findings of this study are presented in section 4 then, followed by a discussion in section 5. Limitations and future research are presented in section 6. Last, the conclusion is summarized in section 7.

## 2 Literature review and hypotheses

### 2.1 Work-family conflict

Work/family conflict is defined as a type of inter-role conflict in which the demands of the work and family domains collide in some way (Greenhaus and Beutell, 1985; Smith et al., 2018; Stoeva et al., 2002). Greenhaus and Beutell (1985) classified work-family conflict into two categories: work interferes with family (WIF) and family interferes with work (FIW). WIF is defined as a conflict of roles between work and family caused by work interfering with family members' respective roles (Wu et al., 2018). The individuals believe that they are allocating all the resources available to perform the task in the workplace. These jobs and demands divert attention and energy away from family (Yustina and Valerina, 2018). FIW refers to a role conflict between family and work in which the family considers intervening in individual roles at work (Wu et al., 2018). In comparison to WIF, individuals perceive high demands from family and eventually interfere with their roles and obligations in the workplace. For instance, individuals arrive late for work due to the need to care for their children (Yustina and Valerina, 2018).

### 2.2 Job stress

Myriad investigations have been undertaken to delve deeper into the issue of job stress. Multiple studies have also developed various methods for job stress, one of which is the transactional approach. A transactional perspective emphasizes the interaction between individuals and their work environment during the development of stress. This approach entails the Lazarus and Folkman models (1984). In this approach, stress is regarded as an integral part of interaction or interaction between persons and their environment (Mansour and Tremblay, 2018). Several studies defined job stress as an individual’s awareness or perception of personal dysfunction because of work-related conditions or events (Bolino and Turnley, 2005; Parker and DeCotiis, 1983; Sulistiawan et al., 2020). There are two important factors emphasized in this definition. First, job stress is an awareness or emotion that signals that the new condition is considered stressful based on the individual’s perception. Second, the expression of personal dysfunction implies that the conscious experience of the situation is dysfunctional, resulting in discomfort for the individual. Thus, job stress is no longer defined in terms of a trigger or an outcome of work stress but rather in terms of uncomfortable feelings experienced by individuals.

### 2.3 Workplace deviant behavior

Based on established studies, deviant behavior is referred to by a variety of labels, including counterproductive behavior (Oh et al., 2014), organizational misbehavior (Thomas and Harris, 2021), noncompliant behavior (Merkle et al., 2020), and anti-citizenship behavior (Ball et al., 1994; Jelinek and Ahearne, 2010), but they all refer to the same thing. Workplace deviance behavior (WDB) is described as voluntary activity by employees that breaches organizational norms and jeopardizes the organization’s and its members' well-being (Fan et al., 2021; Robinson and Bennett, 1995). Robinson and Bennett (1995) introduced the notion of WDB, which is defined as voluntary behavior that breaches organizational norms and poses a threat to the organization’s or members' welfare, or even both. WDB refers to a deviant behavior that employees engage involuntarily because of a lack of ability to conform or because of being motivated to violate to engage in deviant behavior.

WDB is a voluntary activity that can jeopardize the well-being of the organization, its members, or both (Jelinek and Ahearne, 2010). This definition captures several critical points. To begin, WDB actors are members of an organization or employees of an organization. This implies that WDB is performed by members of the organization, not by anyone from outside the company or organization, and not by terminated employees (Jelinek and Ahearne, 2006). Second, WDB is behavior that members of an organization engage in freely. Thus, WDB is conducted by the employee’s free will. Third, WDB jeopardizes organizational norms, procedures, and formal regulations. This is what distinguishes WDB from unethical conduct: WDB is concerned with behavior that violates organizational standards, whereas unethical behavior is concerned with right or wrong behavior as determined by the context of justice, law, or community-adopted behavioral principles (Brock Baskin et al., 2021; Robinson and O'Leary-Kelly, 1998). Fourth, because WDB is defined as an activity that poses a threat to the organization or its members, small violations are excluded.

Darrat et al. (2017) argued that organizational, interpersonal, and customer-directed deviance are all manifestations of deviant behavior. Organizational deviance is defined as behavior that is opposed to accepted norms and is directed against the organization (Jelinek and Ahearne, 2006). According to Robinson and Bennett (1995), organizational deviance is the voluntary activity by employees that jeopardizes the organization’s well-being. Organizational deviation has a substantial impact on the firm’s bottom line, as nearly every part of the organization is targeted in some way, including staff theft.

Theoretically, interpersonal deviance is a critical topic to examine since interpersonal deviance has been shown to impair performance in business units (Dunlop and Lee, 2004). Bennett and Robinson (2000) assert that interpersonal deviance can impair both individual and organizational effectiveness. Interpersonal deviant behavior can be detrimental to others, especially members of the organization (Aquino et al., 1999; Bennett and Robinson, 2000). Interpersonal deviance, according to Bennett and Robinson (2000), is deviant behavior directed against coworkers in an organization, such as disparaging someone at work, acting rudely toward coworkers, or pranking coworkers at work. According to Mahmood et al. (2020), interpersonal deviance is a norm-defying activity that undermines organizational norms, and the direct consequence of deviant interpersonal behavior is felt directly by organizational members. Being disrespectful to a coworker, humiliating a coworker, or saying hurtful things to a coworker are all manifestations of interpersonal deviance.

Front-line deviance is defined as the behavior demonstrated to clients/customers through complaints about corporate standards and violations of client protocols such as dress codes or office rules (Jelinek and Ahearne, 2006, 2010). Jelinek and Ahearne (2010) note that front-line deviance can be demonstrated by complaining to clients about the workload, which creates a negative impression of the company in the minds of clients, jeopardizing the organization’s ability to build positive relationships with customers.

### 2.4 Work-family conflict and job stress

According to Hobfoll’s spiral of resource loss, individuals have limited resources and struggle to manage their work and familial demands, leading to WIF (Mansour and Commeiras, 2015) or FIW. According to COR theory, salespeople who are subjected to stressful and difficult work conditions frequently lose valuable resources. As a consequence, according to Hobfoll’s concept of the spiral of loss of resources, they have limited resources and struggle to manage their professional and family duties, resulting in WFC (Mansour and Commeiras, 2015) or FWC. Mansour and Tremblay (2018) further justify this spiral by asserting that resource depletion can result in negative emotions, which can lead to a decline in emotional well-being. Regarding the available resources, the conservation of resource model posits that an individual with limited resources may be more susceptible to subsequent losses and that an early loss may result in later losses (Hobfoll, 2001; Mansour and Tremblay, 2018). Consequently, the WFC and FWC may establish a new potential source of resource loss, resulting in stress and burnout (Karatepe et al., 2010; Rabenu et al., 2017; Smith et al., 2018). Thus,

H1a WIF positively affects job stress

H1b FIW positively affects job stress

### 2.5 Job stress and workplace deviant behavior

Job stress arises when one’s resources are depleted, and one has physical and psychological responses, resulting in an inability to fulfill job demands (Lazarus and Folkman, 1984; Swimberghe et al., 2014). Such stress is commonly associated with job demands, including anxiety, pressure, and exhaustion (Good and Schwepker, 2022). Although some stress, known as eustress, benefits individuals by enhancing the effectiveness of physical, cognitive, or demanding work, job stress can be detrimental if left unchecked.

Several theories can be applied to explain the association between job stress and workplace deviant behavior. First, affective events theory (AET) emphasizes workplace events as the source of emotional states and explains how effective reactions might influence one’s assessment or happiness with the job to influence behaviors at work (Raza et al., 2017; Weiss and Cropanzano, 1996). According to AET, deviant workplace behavior is considered affect-driven conduct that occurs as a reaction to workplace emotion. Second coping theory by Lazarus and Folkman (1984). Swimberghe et al. (2014) argued that coping entails modifications to one’s cognitive and behavioral mechanism for coping with severe demands on one’s personal resources. Coping, in this sense, refers to the techniques or behaviors used by sales personnel to handle job stress. The withdrawal from non-work and/or work obligations by salespeople, which is depicted by absenteeism, is one such method. Absenteeism may also provide temporary respite but may increase potential work demands, thereby perpetuating a downward spiral of declining sales (Junaedi and Wulani, 2021; Swimberghe et al., 2014). These behaviors aim to change the work environment to alleviate employee role conflict. Thus,

H2a Job stress positively affects organizational deviant behavior

H2b Job stress positively affects interpersonal deviant behavior

H2c Job stress positively affects front-line deviant behavior

### 2.6 The mediating role of job stress

Employees who experience a high level of work-family conflict will experience resource loss and will act negatively in the workplace (Chen et al., 2020; Hobfoll, 2001). Employees who suffer a high level of work-family conflict may feel greater stress and receive less support from their partners. Consequently, they may respond negatively toward their coworkers and their work and may become less effective in resolving workplace conflicts (Rubab, 2017). As a result, they may be unable to engage with their customers nicely, developing a more hostile perception about their company, coworkers, and customers, and therefore participating in workplace deviant behavior. Thus, it is considered that if an employee is confronted with work-family conflict, this results in stress, which results in deviant workplace behavior. Thus,

H3a Job stress mediates the relationship between WIF and workplace deviant behavior (organizational, interpersonal, and front-line)

H3b Job stress mediates the relationship between FIW and workplace deviant behavior (organizational, interpersonal, and front-line)

## 3 Research Method

### 3.1 Sample and data collection

To fulfill the study’s research objectives, we conducted an online survey of salespeople currently employed in business-to-business or business-to-customer sales. We collected data from 321 respondents in total. The resulting sample included salespeople from a variety of industries: 52.2 percent worked for service firms, while 47.8 percent worked for manufacturing firms. 62.6 percent of respondents are male, and the average respondent is 26 years old and has four years of professional sales experience. Ethical approval was granted, and consent was obtained from all the respondents in this study.

Respondents were asked to report sensitive behavior such as deviance in this study, and the research team addressed this by taking several phases. To begin, we structured surveys thoroughly to eliminate the threat of certain items that have been shown to decrease reporting of low base rates (Peterson, 2002). Second, prior research has demonstrated that both voluntarism and confidentiality decrease respondents' reluctance to disclose information (Aquino et al., 1999; Jelinek and Ahearne, 2010). As such, our email invitation emphasizes that participation is entirely voluntary and confidential. Third, our surveys are completely anonymous; respondents provide no identifying information when submitting their responses; Robinson and Bennett (1995) argue that this is necessary to elicit reporting of deviant behavior. Fourth, regarding the survey’s sensitive measure, respondents were asked to report their behavior over the previous year following Robinson and O'Leary-Kelly (1998) recommendation that a 12-month retrospective time frame can facilitate respondents disclosing information about the survey’s sensitive behavior. Finally, prior research has demonstrated that computer-administered questionnaires can assist in overcoming the low level of data collection on sensitive measures. As a result, we conducted our survey online rather than using traditional pencil and paper formats (Jelinek and Ahearne, 2006).

Universitas Airlangga, as represented by its research department, had granted ethical approval for this research. The research center at Universitas Airlangga, Development and Innovation Institute for Publishing Journals and Intellectual Property Rights, is accountable for managing publications, research papers, innovation, and rights in intellectual property. This research center is responsible for conducting research and supervising the creation of novel research products for the public benefit. Moreover, it can grant ethical approval to studies performed by academics at Universitas Airlangga.

### 3.2 Measures

All measures used a response scale ranging from 1 to 7, with one indicating “strongly disagree”, and seven indicating “strongly agree”. This measurement scale was previously used in several studies. Prior to distributing the survey instrument to respondents, the English version was translated into Bahasa. Work-family conflict, which is comprised of WIF and FIW, was assessed using six items from Carlson et al. (2000). Jelinek and Ahearne’s (2006) four-item scale was used to assess organizational deviance. We employed five-item scale items from Jelinek and Ahearne (2006) to assess interpersonal deviance. Front-line deviance was measured by using five items from Jelinek and Ahearne (2006). Job stress was measured using Parker and DeCotiis (1983) eight items questionnaire.

### 3.3 Data analysis

SmartPLS 3.3.6 was used to test the proposed hypotheses in this study. PLS was chosen because it is capable of handling complex structural models with a large number of constructs, indicators, mediation mechanisms, and also moderating effects (Hair et al., 2016). PLS is a two-step procedure. To begin, we evaluated the measurement model to ensure its validity and reliability. Second, the hypotheses proposed were analyzed. Because all variables were self-rated, common method variance (CMV) was evaluated using a full collinearity test before conducting the formal PLS analysis. Kock and Lynn (2012) proposed calculating the variance inflation factor (VIF) to detect CMV infection; a value greater than 3.3 indicates that the constructs are infected. Our findings indicated that the VIF scores for the constructs ranged from 1.21 to 2.89. As a result, we can confirm that our study was CMV-free.

## 4 Results

### 4.1 Validity and reliability

**Figure 1:**
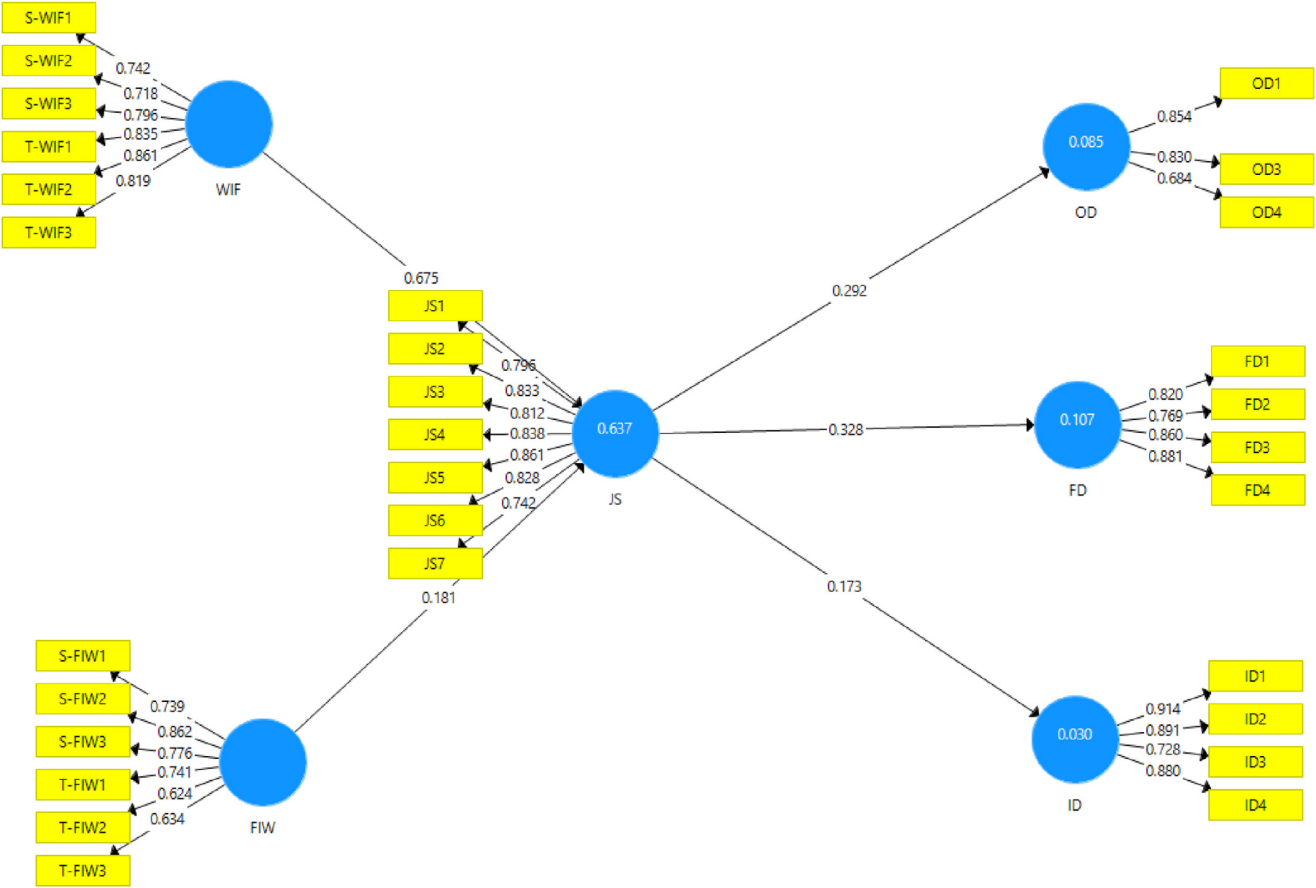
PLS structural path.

Three types of analyses were applied to the measurements in determining the quality of the outer model. To begin, verify convergent validity by analyzing the outer loading score in each indicator and the average variance extracted (AVE) using a 0.50 cut-off value (Hair et al., 2016). Some indicators were eliminated in the first run due to low outer loading scores (ID5, OD2, JS8). After eliminating the indicators with low loading scores, all the indicators met the criteria. As shown in Table 1 and Figure 1, the loading factor score and AVE both meet the criteria greater than 0.50.

**Table 1:** Outer model assessment.

Variables	Indicators	Outer loading	Cronbach’s Alpha	Composite Reliability	AVE
Frontline deviance behavior	FD1	0.820	0.857	0.901	0.695
	FD2	0.769			
	FD3	0.860			
	FD4	0.881			
Interpersonal deviant behavior	ID1	0.914	0.881	0.916	0.734
	ID2	0.891			
	ID3	0.728			
	ID4	0.880			
Job stress	JS1	0.795	0.916	0.921	0.667
	JS2	0.833			
	JS3	0.812			
	JS4	0.838			
	JS5	0.861			
	JS6	0.828			
	JS7	0.742			
Organizational Deviant	OD1	0.854	0.705	0.834	0.629
	OD3	0.830			
	OD4	0.684			
Family interfere work	S-FIW1	0.739	0.830	0.874	0.639
	S-FIW2	0.862			
	S-FIW3	0.876			
	T-FIW1	0.741			
	T-FIW2	0.624			
	T-FIW3	0.634			
Work interferes family	S-WIF1	0.742	0.884	0.912	0.635
	S-WIF2	0.718			
	S-WIF3	0.796			
	T-WIF1	0.835			
	T-WIF2	0.861			
	T-WIF3	0.819

Second, test for consistency within the indicator by analyzing the composite reliability score for each data set. If the variables have a composite reliability score greater than 0.70, they are considered reliable (Hair et al., 2016). Finally, discriminant validity was assessed using both the Fornell-larcker and Henseler et al. (2015) recommendations, namely the heterotrait-monotrait correlations ratio (HTMT) criterion. An HTMT value higher than 0.90 implies a deficiency in discriminant validity (Hair et al., 2016; Henseler et al., 2015). As shown in Table 2, none of the HTMT values were greater than 0.90, implying that the variables under consideration were discriminant. Additionally, the AVE scores for all variables exceed the correlation. Thus, we can confirm that this study met both convergent and discriminant validity requirements.

**Table 2:** Fornell-larcker and HTMT.

	1	2	3	4	5	6
FD	**0.834**	*0.264*	*0.818*	*0.353*	*0.638*	*0.312*
FIW	0.225	**0.734**	*0.185*	*0.644*	*0.445*	*0.685*
ID	0.692	0.131	**0.856**	*0.185*	*0.638*	*0.167*
JS	0.328	0.591	0.173	**0.816**	*0.363*	*0.857*
OD	0.477	0.361	0.483	0.292	**0.793**	*0.322*
WIF	0.290	0.608	0.156	0.785	0.262	**0.797**

*Notes:* FD = Frontline deviance, FIW = Family interfere work, ID = Interpersonal deviance, JS = Job stress, OD = Organizational deviance, WIF = Work interfere family. The diagonal values in **bold** are the square root of AVE. The values in *italic* are the HTMT scores.

### 4.2 Hypothesis testing

#### 4.2.1 Direct effect

To test the proposed hypotheses, we analyzed the path coefficients. We followed the recommendation from Sulistiawan et al. (2022) and assessed the proposed hypotheses' strength using bootstrapping with 5000 subsamples. As Figure 1 and Table 3 demonstrate, WIF and FIW positively affect job stress. Thus, H1a and H1b were supported (β = 0.656, p < 0.001; β = 0.212, p < 0.001). We proposed that job stress has a positive effect on organizational, interpersonal, and front-line deviant behavior. Our results confirm those proposed hypotheses, thus H2a, H2b, H2c were supported (β = 0.286, p < 0.001; β = 0.176, p < 0.05; β = 0.307, p < 0.001).

**Table 3:** Direct effect results.

Structural Path	β	Standard Deviation	p-values	5.00%	95.00%	Remarks
WIF > JS	0.675	0.048	0.000	0.603	0.757	supported
FIW > JS	0.181	0.062	0.002	0.077	0.278	supported
JS > OD	0.292	0.064	0.000	0.175	0.398	supported
JS > ID	0.173	0.091	0.028	0.078	0.316	supported
JS > FD	0.328	0.077	0.000	0.208	0.458	supported

*Notes:* FD = Frontline deviance, FIW = Family interfere work, ID = Interpersonal deviance, JS = Job stress, OD = Organizational deviance, WIF = Work interfere family.

#### 4.2.2 Indirect effect

Concerning the mediating impact, the Zhao et al. (2010) approach was used. Zhao et al. (2010) suggested that when the direct impact is significant, the mediating effect is only partial. When the direct impact is not significant, the mediating variable is considered fully mediated. The indirect effect is depicted in Table 4. Before we test the mediating effect, we conducted a direct effect analysis of WIF and FIW on all types of WDB. In H3a, we proposed that job stress mediates the relationship between WIF and all types of WDB. Our results confirm those hypotheses, thus supporting H3a. Our results also confirm that job stress mediates the relationship between FIW and all types of WDB, thus confirming H3b.

**Table 4:** Indirect effect results.

Structural path	β	Standard Deviation	p-values	5.00%	95.00%	Remarks
*Direct effect*						
WIF→OD	0.032	0.108	0.297	-0.160	0.188	
WIF→ID	0.024	0.176	0.136	-0.321	0.233	
WIF→FD	0.060	0.135	0.444	-0.161	0.281	
*Indirect effect*						
WIF > JS > OD	0.188	0.040	0.000	0.134	0.263	Supported (Fully mediated)
WIF > JS > ID	0.115	0.055	0.018	0.065	0.210	Supported (Fully mediated)
WIF > JS > FD	0.201	0.051	0.000	0.137	0.303	Supported (fully mediated)
*Direct effect*						
FIW→OD	0.036	0.096	0.001	0.136	0.469	
FIW→ID	0.040	0.158	0.400	-0.179	0.303	
FIW→JD	0.304	0.125	0.388	-0.170	0.221	
*Indirect effect*						
FIW > JS > OD	0.061	0.024	0.007	0.027	0.110	Supported (Partially mediated)
FIW > JS > ID	0.037	0.022	0.047	0.015	0.082	Supported (Fully mediated)
FIW > JS > FD	0.065	0.027	0.007	0.028	0.113	Supported (Fully mediated)

Notes: FD = Frontline deviance, FIW = Family interfere work, ID = Interpersonal deviance, JS = Job stress, OD = Organizational deviance, WIF = Work interfere family.

## 5 Discussions

This study provides a comprehensive analysis of the drivers of workplace deviant behavior. This study examines the effect of work-family conflict, which consists of WIF and FIW, on job stress. In addition, this study investigates how job stress affects three types of WDB (organizational, interpersonal, and front-line deviant behavior). Furthermore, this study investigates the mechanism of job stress in mediating the relationship between work-family conflict and three types of WDB.

The results of this study corroborate prior studies' findings. First, this study confirms that work-family conflict becomes the source of job stress. In other words, when a salesperson has a high level of work-family conflict, this will result in the salesperson experiencing a high level of job stress. Earlier research has revealed that both WIF and FIW are key antecedents in predicting employee job stress levels, and this finding is consistent (Amiruddin, 2019; Mansour and Tremblay, 2018). The findings of this study also support the spiral of resource loss phenomena, which claims that individuals have a variety of roles to fulfill and that they have limited resources to fulfill these tasks. Individuals who struggle to meet the expectations of their roles, both at work and home, will experience resource depletion, which will result in greater perceived stress by the individual. The positive effect of FIW on job stress also confirms that work-family conflict phenomena have a spillover effect. According to the notion of spillover, as stated by Staines (1980), attitudes and behaviors can be transmitted from work life to home life. For example, if employees are content with their jobs, they will be satisfied with their lives outside of work and vice versa.

Second, job stress becomes a significant predictor of why employees engage in counterproductive behavior. These findings corroborate previous research indicating that high levels of work stress encourage employees to engage in deviant behavior (Junaedi and Wulani, 2021; Swimberghe et al., 2014). Moreover, salespeople may attempt to alleviate job stress by participating in deviant behavior both outside the firm and with coworkers. Job stress can diminish sales associates' cognitive and emotional resources, resulting in bad customer encounters (Swimberghe et al., 2014). Boundaries define the nature of retail sales for a particularly sensitive position within retail businesses. Front-line sales staff are required to interact with customers daily, which can result in significant role stress (Darrat et al., 2017). Employees that are stressed are overwhelmed by their resources and become fixated on the source of their tension and their situation. They can minimize their efforts to conserve the remaining resources. Another possibility is that they exhibit excessive performance in front of consumers as a result of their exhaustion (Junaedi and Wulani, 2021). The findings of this study can also be justified by the social exchange theory, which states that individuals deviate from the norm to reciprocate with the organization, coworkers, and customers who are perceived to be the source of their distress. According to the sample in this study, front-line personnel may face job stress because of high job requirements and client interaction requirements. This kind of work needs them to have a thorough understanding of the product and a certain level of service to please consumers and enhance corporate sales. Front-line employees that are stressed out display higher deviant conduct toward the firm and its clients.

Third, our study confirms that job stress has a mediating mechanism in the relationship between work-family conflict and three types of WDB. Salespeople frequently draw on the same cognitive and emotional resources in stressful work circumstances as they do in their family roles. Patience and understanding are also crucial resources for developing the interpersonal relationships required for a successful buyer-seller partnership (Swimberghe et al., 2014). The findings of this study suggest that job stress is a mediating factor in the link between work-family conflict and salesperson deviation since front-line staff deal with client complaints/problems and/or fail to close sales most of the time. As a result, it is reasonable to speculate that job stress acts as a mediator between work-family conflict and salesperson deviant behavior. High levels of work-family conflict can wreak havoc on a partner’s coping mechanisms, eventually resulting in stress. The more depleted the sales force’s resources, the more likely they will encounter job stress. This job stress is what drives salespeople to engage in deviant behavior in an attempt to atone for what they have experienced.

## 6 Practical implications

Our study has several important practical implications for managers and organizations. First, organizations must continue to assist, cultivate positive relationships with, and involve employees in decision-making processes to prevent job stress and deviant behavior. Moreover, because individual differences might influence how workers respond to on-site pressure, firms must disclose information about company behavior and the work environment. Furthermore, organizations must consider contextual variables that can exacerbate an employee’s unpleasant experience, such as managers' and coworkers' behavior. In this instance, they must provide a safe social atmosphere and provide training to ensure that coworkers and superiors realize the consequences of their negative behavior.

Second, organizations can establish policies that help reduce WFC levels, such as family-friendly practices that demonstrate support for salespeople’s family member roles. Coaching, daycare, family leave, flexible work hours, and on-site job counseling for work-family problems can all boost an organization’s positive image in the minds of employees. These initiatives not only alleviate work/family conflict but also boost the employees' satisfaction with the organization.

Third, managers should evaluate the sales targets assigned to their salespeople. The intense competition across industries should not justify an unjustified increase in sales targets. Setting inappropriate sales targets can result in job stress. To help counteract this detrimental effect, managers may implement a proportional and motivating compensation system that rewards customer relationship management via metrics such as customer satisfaction. Following that, managers can collaborate with salespeople to determine which areas of the job trigger the most work/family conflict. Then, strategies can be implemented to alter work activities to minimize family conflict while maintaining good work standards.

### 6.1 Limitations and future studies

Despite its important contribution to the deviant behavior body of knowledge, this study has several limitations. First, this study only uses one single country. To enhance the generalizability of this study’s findings, future research may replicate the current study’s framework with different sample settings and conduct study comparisons between countries. Second, this study overlooked some potential contextual variables that may diminish the negative effect of WFC and job stress on deviant behavior. Future studies may add moderating variables such as type of personality or regulatory focus mechanism that may diminish the negative effect of WFC and job stress on deviant behavior. In addition, future studies may compare the difference between gender groups because males and female have a different perspective on experiencing WFC and FWC. Furthermore, future studies may consider adding sociodemographic variables (age, tenure, job position, etc) as control variables. Third, even though we have certain procedures for minimizing social desirability bias, future studies may employ other methods of data collection.

## 7 Conclusions

The objective of this study was to examine the drivers of workplace deviant behavior. Specifically, we examine WFC, which consists of WIF and FIW towards job stress. In addition, we also examine the mediating mechanism of job stress in the link between WFC and deviant behavior. We tested our hypotheses by employing PLS-SEM after ensuring the validity and reliability of our data. The findings of this study confirm all hypotheses and support prior studies. The findings of this study are valuable to both academics and organizations. This study was conducted in an area of sales that has received little attention. This study contributes to the literature by focusing on the physical and psychological health of a strained salesman, whereas past research focused on job stress as a stressor. Moreover, this study adds to the body of knowledge on counterproductive work behavior by demonstrating that job stress acts as a moderator in the association between WFC and all types of deviant conduct.

## Declarations

### Author contribution statement

Yu-Te Tu: Contributed reagents, materials, analysis tools or data; Wrote the paper.

Jovi Sulistiawan: Conceived and designed the experiments; Performed the experiments; Analyzed and interpreted the data; Contributed reagents, materials, analysis tools or data; Wrote the paper.

Dian Ekowati: Analyzed and interpreted the data; Contributed reagents, materials, analysis tools or data; Wrote the paper.

Hanif Rizaldy: Performed the experiments.

### Funding statement

This research did not receive any specific grant from funding agencies in the public, commercial, or not-for profit sectors.

### Data availability statement

Data will be made available on request.

### Declaration of interest’s statement

The authors declare no conflict of interest.

### Additional information

No additional information is available for this paper.

## Appendix A Supplementary data

Questionnaire items Questionnaire items
