# Supplementary material for: Work-family conflict and salespeople deviant behavior: the mediating role of job stress
Source: Heliyon. 2022 Oct 7;8(10):e10881. doi: 10.1016/j.heliyon.2022.e10881 (PMC9552108; doi:10.1016/j.heliyon.2022.e10881)
Supplement: Questionnaire items [file mmc1.docx]

**Work interferes family (Carlson et al., 2000)**

1. My work keeps me from my family activities more than I would like.

2. The time I must devote to my job keeps me from participating equally in household

responsibilities and activities.

3. I must miss family activities due to the amount of time I must spend on work

responsibilities.

4. When I get home from work, I am often too frazzled to participate in family activities/

responsibilities.

5. I am often so emotionally drained when I get home from work that it prevents me from

contributing to my family.

6. Due to all the pressures at work, sometimes when I come home, I am too stressed to do the

things I enjoy.

**Family interferes work (Carlson et al., 2000)**

1. The time I spend on family responsibilities often interferes with my work responsibilities.

2. The time I spend with my family often causes me not to spend time on activities at work

that could be helpful to my career.

3. I must miss work activities due to the amount of time I must spend on family

responsibilities.

4. Due to stress at home, I am often preoccupied with family matters at work.

5. Because I am often stressed from family responsibilities, I have a hard time concentrating

on my work.

6. Tension and anxiety from my family life often weakens my ability to do my job

**Job Stress (Parker and DeCotiis, 1983)**

1. Working here makes it hard to spend enough time with my family
2. I spend so much time at work, I cannot see the forest for the trees
3. Working here leaves little time for other activities
4. I frequently get the feeling I am married to the company
5. I have too much work and too little time to do it in
6. I sometimes dread the telephone ringing at home because the call might be job-related
7. I feel like I never have a day off
8. Too many people at my level in the company get burned out by job demands

**Organizational Deviance (Jelinek & Ahearne, 2006)**

1. "Fudged" an expense report.
2. Ran personal errands when they should have been doing work.
3. Used company resources (paper, supplies, fax, copier) for personal purposes.
4. Put off work-related duties to attend to personal things

**Interpersonal Deviance (Jelinek & Ahearne, 2006)**

1. Accepted credit for the work of other people.
2. Cursed at coworkers and colleagues.
3. Criticized coworkers and colleagues.
4. Blamed coworkers and colleagues when things went wrong at work.
5. Said hurtful things to coworkers and colleagues.

**Frontline Deviance (Jelinek & Ahearne, 2006)**

1. Told customers some of the things that are bothersome about your company.
2. Used deceptive tactics while selling to prospects or customers.
3. Complained to friends and family about company.
4. Made our company look bad to people who do not work here.
